# Prefronto-striatal physiology is associated with schizotypy and is modulated by a functional variant of *DRD2*

**DOI:** 10.3389/fnbeh.2014.00235

**Published:** 2014-07-09

**Authors:** Paolo Taurisano, Raffaella Romano, Marina Mancini, Annabella Di Giorgio, Linda A. Antonucci, Leonardo Fazio, Antonio Rampino, Tiziana Quarto, Barbara Gelao, Annamaria Porcelli, Apostolos Papazacharias, Gianluca Ursini, Grazia Caforio, Rita Masellis, Artor Niccoli-Asabella, Orlando Todarello, Teresa Popolizio, Giuseppe Rubini, Giuseppe Blasi, Alessandro Bertolino

**Affiliations:** ^1^Department of Basic Medical Science, Psychiatric Neuroscience Group, Neuroscience and Sense Organs, University of Bari Aldo MoroBari, Italy; ^2^IRCCS “Casa Sollievo della Sofferenza”, San Giovanni RotondoFoggia, Italy; ^3^Department of Behavioural Sciences, Cognitive Brain Research Unit, University of HelsinkiHelsinki, Finland; ^4^Lieber Institute for Brain Development, Johns Hopkins University Medical CampusBaltimore, MD, USA; ^5^Department of Internal Medicine and of Public Medicine, Nuclear Medicine Unit, University of Bari Aldo MoroBari, Italy; ^6^pRED, NORD DTA, Hoffmann-La Roche, Ltd.Basel, Switzerland

**Keywords:** schizotypy, dopamine, DRD2, fMRI, SPECT

## Abstract

“Schizotypy” is a latent organization of personality related to the genetic risk for schizophrenia. Some evidence suggests that schizophrenia and schizotypy share some biological features, including a link to dopaminergic D2 receptor signaling. A polymorphism in the D2 gene (*DRD2* rs1076560, guanine > thymine (G > T)) has been associated with the D2 short/long isoform expression ratio, as well as striatal dopamine signaling and prefrontal cortical activity during different cognitive operations, which are measures that are altered in patients with schizophrenia. Our aim is to determine the association of schizotypy scores with the *DRD2* rs1076560 genotype in healthy individuals and their interaction with prefrontal activity during attention and D2 striatal signaling. A total of 83 healthy subjects were genotyped for *DRD2* rs1076560 and completed the Schizotypal Personality Questionnaire (SPQ). Twenty-six participants underwent SPECT with [^123^I]IBZM D2 receptor radiotracer, while 68 performed an attentional control task during fMRI. We found that rs1076560 GT subjects had greater SPQ scores than GG individuals. Moreover, the interaction between schizotypy and the GT genotype predicted prefrontal activity and related attentional behavior, as well as striatal binding of IBZM. No interaction was found in GG individuals. These results suggest that rs1076560 GT healthy individuals are prone to higher levels of schizotypy, and that the interaction between rs1076560 and schizotypy scores modulates phenotypes related to the pathophysiology of schizophrenia, such as prefrontal activity and striatal dopamine signaling. These results provide systems-level qualitative evidence for mapping the construct of schizotypy in healthy individuals onto the schizophrenia continuum.

## Introduction

Schizophrenia is strongly heritable. The risk for schizophrenia can be represented by a continuous Gaussian distribution in the general population (Plomin, 1999) with an arbitrary threshold to distinguish patients and healthy individuals (DSM-V diagnostic criteria) (Bhati, 2013). In light of this, people falling within this distribution and below the threshold still have some theoretical risk for the disease but do not manifest it. A corollary of this perspective is that individuals below the diagnostic threshold also share some of the genetic risk for the disorder (Plomin, 1999).

“Schizotypy” is a latent organization of personality including both positive and negative attenuated symptoms of schizophrenia (Meehl, 1989). Higher rates of schizotypal traits have been found in first-degree relatives of patients with schizophrenia compared with healthy subjects, suggesting that schizotypy is related to a genetic risk for the disorder (Kety et al., 1994; Kendler et al., 1995; Tsuang et al., 1999; Vollema et al., 2002; Ettinger et al., 2014). Nonetheless, variable schizotypy has been found in non-psychiatric individuals (Chen et al., 1997; Croft et al., 2001; Fonseca-Pedrero et al., 2007, 2008, 2009, 2011; Aguirre et al., 2008; Kwapil et al., 2008; Noguchi et al., 2008; Wilson et al., 2008; Bedwell et al., 2009), suggesting that this trait may be studied in healthy subjects without the confounds and limitations commonly found in patients.

Schizotypy has been correlated with neurocognitive, behavioral, and social deficits, which are qualitatively similar to those found in patients with schizophrenia but quantitatively less severe (Croft et al., 2001; Noguchi et al., 2008; Wilson et al., 2008; Bedwell et al., 2009; Giakoumaki, 2012). For example, deficits in cognitive flexibility, working memory, and prepulse inhibition have been consistently reported in healthy subjects with high schizotypy (Giakoumaki, 2012). These findings suggest that risk for schizophrenia, schizotypy, and cognitive abnormalities may be interconnected. Indeed, previous studies have demonstrated that dopamine and D2 receptor are implicated both in schizotypy and risk for schizophrenia, as well as in schizophrenia-related phenotypes, including attentional deficits and associated brain physiology (Zhang et al., 2007; Delawalla et al., 2008; Blasi et al., 2010). In this context, consistent evidence suggests abnormal dopamine signaling in the striatal regions (Abi-Dargham et al., 1998, 2000) particularly in the putamen (Soret et al., 2003; Scherfler et al., 2005; Kegeles et al., 2010; Suridjan et al., 2013) of patients with schizophrenia and of subjects at high risk for this brain disorder. Furthermore, striatal dopamine release positively correlated with schizotypal personality traits in healthy subjects in a PET study (Woodward et al., 2011). A recent single-photon emission computed tomography (SPECT) investigation revealed that the availability of striatal D2 receptors is associated with schizotypal features in healthy volunteers (Chen et al., 2012). Finally, another study in patients with schizotypal personality disorder (SPD) indicated exaggerated dopamine release in the striatum following *d*-amphetamine challenge (Abi-Dargham et al., 2004). However, more investigation of the function of brain networks is needed to map schizotypy in healthy individuals onto the Gaussian distribution of schizophrenia risk.

Genetic risk for schizophrenia has been widely investigated in a number of association studies, but there is still little evidence confirming the association of the dopamine D2 receptor (*DRD2*) gene with the disease (Allen et al., 2008). More recent data from the Psychiatric Genomic Consortium seem to indicate a strong association between *DRD2* and schizophrenia in the largest sample studied to date (Ripke et al. oral communication, World Congress Psychiatric Genetics, Boston 2013). Moreover, some of the strongest candidate genes for schizophrenia, including ZNF804A, DISC1, and DTNBP1, have well-known biological effects on D2 receptors or D2 signaling partners (Marley and von Zastrow, 2010; Girgenti et al., 2012; Papaleo et al., 2012). Also, D2 receptors have consistently been implicated in the pharmacodynamic mechanism of antipsychotic drugs (Carpenter and Koenig, 2008), which are the only medications with established effectiveness in schizophrenia (Carpenter and Koenig, 2008).

The *DRD2* gene is transcribed into two main mRNA isoforms, resulting in as many proteins: D2 short (S) and D2 long (L). D2L receptors mainly mediate post-synaptic signaling, whereas D2S receptors mainly serve as auto-receptors on pre-synaptic neurons (Usiello et al., 2000). The intronic *DRD2* polymorphism rs1076560 (guanine > thymine (G > T)) is associated with mRNA splicing (Zhang et al., 2007). More specifically, the minor (T) allele is associated with a lower ratio of the expression between D2S and D2L in the post-mortem prefrontal cortex and striatum, as well as with altered activity of the striato-thalamic-prefrontal pathway during tasks probing different brain functions (Zhang et al., 2007; Bertolino et al., 2009a,b, 2010; Blasi et al., 2009, 2011). More recently, we have demonstrated that the effect of this single nucleotide polymorphism (SNP) on prefrontal cortical activity during cognition may also be associated with indirect modulation of dopamine D2 signaling via the striatum (Bertolino et al., 2010). Importantly, the association between dopamine-related genetic variation and brain function shows its impact on some core phenotypes of schizophrenia, such as attentional processing and related cingulate and prefrontal activity (Blasi et al., 2005, 2007, 2010, 2011; Delawalla et al., 2008; Weickert et al., 2009). Altogether, this evidence strongly suggests a relationship between D2 receptor genetic variability, attentional processes, and risk for schizophrenia. Notably, schizotypy is also associated with deficits in attention. In fact, two different studies have reported a correlation between this trait and behavioral abnormalities during attentional processing (Breeze et al., 2011; Giakoumaki, 2012). However, to our knowledge, there has been no functional imaging study investigating the relationship between schizotypy and brain activity during attention, as well as its putative interaction with the *DRD2* gene.

Based on this evidence, we aim to:

1. Determine the association of schizotypy scores with *DRD2* rs1076560 genotype in healthy individuals.
2. Investigate how schizotypy and the *DRD2* rs1076560 genotype interact on prefrontal activity during attention and D2 striatal signaling in healthy subjects.

## Materials and methods

### Subjects

A total of 83 healthy subjects were recruited in this study. All participants were Caucasians from the region of Puglia, Italy. Table 1 shows details about the demographics of the sample, while Table 2 shows information about subjects that participated in multiple experiments. All subjects were evaluated with the Structured Clinical Interview for the Diagnostic and Statistical Manual of Mental Disorders (First et al., 1996), 4th Edition, to exclude any psychiatric disorder. Along with the absence of psychiatric conditions, other exclusion criteria were represented by a history of significant drug or alcohol abuse, active drug use in the previous year, head trauma with loss of consciousness, and any significant medical condition. The present experimental protocol was approved by the local institutional review board at the Policlinico of Bari, Bari, Italy. After giving the subjects a thorough description of the study, written informed consent was obtained. All subjects underwent one or more of the procedures described below.

**Table 1:** **Demographics of the subjects included in the study**.

	***N***	Age*	**Sex**	HollingShead*	Handedness*	QI*	SPQ*
Association of SPQ scores with rs1076560							
GG	42	27.7 ± 5.8	20 m	33.2 ± 15	0.7 ± 0.5	107.9 ± 14.4	7.5 ± 4.9
GT	25	25.4 ± 5.5	6 m	35.3 ± 12.4	0.8 ± 0.2	103.6 ± 8.4	11.7 ± 7.7
fMRI							
GG	55	27.2 ± 5.3	20 m	38.4 ± 17.3	0.74 ± 0.4	112.5 ± 12.4	7.98 ± 5.9
GT	13	22.9 ± 3	4 m	33.96 ± 15.6	0.63 ± 0.57	106.5 ± 12.9	7.92 ± 4.5
SPECT study							
GG	17	23.6 ± 3.33	8 m	41.4 ± 17.5	0.68 ± 0.4	113.11 ± 15.4	9.7 ± 9.6
GT	9	23 ± 3.3	5 m	38.7 ± 9.8	0.8 ± 0.2	107 ± 16.6	6.1 ± 5.4

*mean ± SD.

**Table 2:** **Number of subjects that participated in multiple experiments and genotype distribution**.

	**Total subjects number**	**GG**	**GT**
Ex1.Association of SPQ scores with rs1076560	67	42	25
Ex2.fMRI	68	55	13
Ex3.SPECT study	26	17	9
Ex1+Ex2	22	18	4
Ex1+Ex3	8	4	4
Ex2+Ex3	10	8	2
Ex1+Ex2+Ex3	3	3	0

### Genotype determination

Subjects were genotyped for *DRD2* rs1076560 in our laboratory through a previously described allele-specific primer PCR approach (Papp et al., 2003; Bertolino et al., 2009a, 2010; Blasi et al., 2009, 2011; Fazio et al., 2011; Sambataro et al., 2013). Briefly, a MiniOpticon 48-well Real Time PCR System (Biorad) was used for PCR amplification and subsequent fluorescence melting curve analysis on a 25-uL volume of PCR reaction. Primers with differential melting temperatures (Tm) were obtained by introducing a random guanine-cytosine (GC) segment at the 5’ end of one specific forward primer. Specifically, the GC clamp was added to the primer of the product with the highest initial Tm in order to obtain a difference of 4°C or more between the two alleles (G *vs*. T).

Forward and reverse primers were titrated over a range of 50–900 nM, and allele-specific reactions were analyzed with individual forward and reverse primer sets. The amplification conditions consisted of a 10-min preincubation at 95°C (activation of the *Taq* DNA polymerase), followed by 40 cycles of denaturation at 95°C for 15 s, and then primer annealing and extension for 1 min at 60°C. The fluorescence melting curve was analyzed immediately after the amplification phase. SYBR Green® fluorescent dye was used to visualize DNA binding using fluorescence melting curves. Consistent with the distribution observed in earlier studies (Zhang et al., 2005), no *DRD2* TT subjects were observed in this sample. The allelic distribution of *DRD2* showed a Hardy Weinberg equilibrium (*df* = 1, χ^2^ = 0.58, *p* = 0.48).

### Schizotypal personality questionnaire

All subjects completed the Schizotypal Personality Questionnaire (SPQ; Raine, 1991, 2006). The SPQ is a 74-item validated self-report questionnaire with a “yes/no” response format that incorporates DSM-III-R criteria (American Psychiatric Association, 1987) for the diagnosis of SPD. The questionnaire consists of nine subscales, which have been found to correspond to three factors: cognitive-perceptual, interpersonal, and disorganized factors. SPQ total scores were used.

### Association of SPQ with *DRD2* rs1076560

A one-way ANOVA with the SPQ total score as the dependent variable and the *DRD2* rs1076560 genotype as the predictor was used to test for the association of schizotypy and genetic variability with rs1076560.

### Association of SPQ and *DRD2* rs1076560 with imaging phenotypes

#### SPECT

Twenty-six healthy subjects (17 GG, 9 GT) underwent SPECT with [^123^I]IBZM radiotracer, which binds to D2 receptors (Kung et al., 1990). Each individual was intravenously injected with an average of 150 MBq (range: 111–186 MBq) of commercially available IBZM radiotracer (GE Healthcare, USA) (Meyer et al., 2008). Potassium iodide solution (Lugol) was administered at least 3 h before and 12 h after radiopharmaceutical injection to block the thyroid uptake of free radioactive iodide. Images were acquired 1.5 h after IBZM injection (Brücke et al., 1991). A dual-head gamma camera (Infinia, GE Healthcare, USA) equipped with parallel-hole low-energy high-resolution collimators was used. SPECT data were acquired using the following parameters: 128 × 128 matrix, a rotation of 360°, 6° view angle, 45 s for projection. The slice thickness was 3.68 mm, the acquisition time was 22 min, and total brain counts of >1 million were achieved in all examinations. Reconstruction was performed by a filtered back projection with a Butterworth filter (cut-off frequency: 0.3 cycle/cm, 10th order) to provide transaxial slices. The attenuation correction was performed according to Chang’s method (attenuation coefficient: 0.12 cm^−1^) after manually drawing an ellipse around the head contour (Tatsch et al., 2002). The system’s spatial resolution (full width at half-maximum) at a radius of rotation of 15.9 cm was 11 mm, as reported elsewhere (Soret et al., 2003). For the analysis of the striatal radiotracer uptake, slices were reoriented parallel to the canthomeatal line.

#### SPECT data processing

The irreversible binding characteristics and the uptake stability of the regional IBZM radiotracer for the D2 receptor have been shown to allow for the estimation of the specific-to-nondisplaceable equilibrium partition coefficient (V3″), which is proportional to the free transporter or receptor density (Bmax) (Laruelle et al., 1994; Frankle et al., 2004). V3″ can be calculated as indicated earlier (Laruelle et al., 1994; Scherfler et al., 2005). Under equilibrium conditions between a compartment with specific binding and a compartment representing nonspecifically bound and free activity, V3″ is proportional to Bmax given that the dissociation constant and the volume of distribution of the nonspecifically bound and free activity compartment (V2) are relatively invariant. The occipital region was selected as the background region because (1) the density of dopamine D2 receptors is negligible compared with the striatum (Lidow et al., 1989); (2) this region can be identified with greater reliability than the cerebellum (Laruelle et al., 1996); and (3) in humans, IBZM activity in the occipital region is equal to the nonspecific activity in the striatum (Seibyl et al., 1992).

Therefore, as in earlier studies (Verhoeff et al., 1993; Laruelle et al., 1996; Beukers et al., 2009), the occipital region was used to model the nonspecifically bound and free activity compartment. V3″ was calculated in all voxels with the formula reported below as in previous studies (Scherfler et al., 2005; Bertolino et al., 2010):

$$\text{V}3^{"}=\left[{\left(\text{counts per minute}/\text{voxel}\right)}_{\text{VT}}-{\left(\text{counts per minute}/\text{voxel}\right)}_{\text{V}2}\right]/{\left(\text{counts per minute voxel}\right)}_{\text{V}2}$$

where VT represents specific binding, and V2 is the nonspecifically bound and free activity compartment. The image transformation, the calculation of V3″, and the statistical analysis were performed using SPM8 (Wellcome Department of Cognitive Neurology, London, UK). V2 was calculated with the ROI of the occipital lobe from WFU PickAtlas software version 1.04 (Functional MRI Laboratory at the Wake Forest University School of Medicine),^1^ (Lancaster et al., 2000; Tzourio-Mazoyer et al., 2002; Maldjian et al., 2003).

Since the parametric images of IBZM V3″ lack anatomical detail, an indirect approach was employed for the spatial normalization, as detailed in previous studies (Rakshi et al., 1999; Scherfler et al., 2005). Briefly, the raw IBZM SPECT data of each subject were normalized on the SPECT template in MNI (Montreal Neurological Institute) space (Scherfler et al., 2005) with a 12-parameter affine transformation of the raw data onto the template image followed by the estimation of the nonlinear deformations between the applied images. A mean image of previously normalized raw data acquisitions was then computed and used as a template image. For each individual SPECT acquisition, a parametric V3″ image was calculated. The raw data image was transformed into the template image, and the resulting transformation parameters were then applied to the parametric V3″ image of the corresponding subject. The spatially normalized parametric images were convolved with a Gaussian kernel (6 × 6 × 6 mm) for smoothing.

Group analysis was performed using a general linear model with the V3″ binding of IBZM as the dependent variable, the *DRD2* genotype as a categorical predictor, and SPQ scores as a continuous predictor. Separate contrasts were thus performed to detect the directionality of the correlations (i.e., positive or negative) associated with psychometric schizotypy for each genotype group. We used a statistical threshold of *p* < 0.05 with the family-wise error small-volume corrected (Genovese et al., 2002) using the whole putamen ROI followed by a voxel-based analysis. Regions of interest were created using WFU PickAtlas software version 1.04.^1^ The results are significant after correction for the total number of voxel-wise comparisons.

#### fMRI

Sixty-eight healthy subjects (55 GG and 13 GT) underwent fMRI while performing the variable attentional control (VAC) task, which elicits increasing demand for attentional control and was identical to the one published in previous studies (Blasi et al., 2005, 2007, 2010, 2011, 2013a,b; Zhang et al., 2007). The VAC task allows for the investigation of brain activity during three levels of attentional control (low, intermediate, high), which were obtained while manipulating both the relative directions of arrows with different sizes and related cue words.

Each stimulus of the VAC task was composed of arrows of three different sizes pointing either to the right or to the left, and small arrows were embedded in medium-sized arrows that were in turn embedded in a large arrow. Subjects were instructed by a cue word (big, medium, or small) displayed above each stimulus to press a button corresponding to the direction of the large, medium, or small arrows (right or left). To increase the level of attentional control required, the direction of the arrows was congruent or incongruent for all three sizes. This resulted in the following conditions:

- Low level of attentional control. All three sizes of arrows were congruent in direction with each other. The cue was the word BIG.
- Intermediate level of attentional control. Two types of stimuli were used, and the big arrow was incongruent in direction to the small and medium arrows in both: the cue was BIG in one of them and SMALL in the other.
- High level of attentional control. Two types of stimuli were used, and the medium-sized arrows were incongruent in direction to the big and small arrows in both: the cue was SMALL in one of them, MEDIUM in the other.
- A simple bold arrow pointing either to the left or to right was used as a sensorimotor control condition.

Subjects were instructed to respond to the task stimuli with the right hand using a button box (with a right button for the “right” response and a left button for the “left” response) and to press the response button as fast and as accurately as possible. Furthermore, they were asked to move their thumb onto a small plastic knob placed between buttons after each response. All subjects were trained on the task before the fMRI session. Each stimulus was presented for 800 ms, and the order of the stimuli was randomly distributed throughout the session. The total number of stimuli was 241: 50 HIGH (25 stimuli of each of the two stimulus types that subtended this level of attentional control), 68 INT (34 stimuli of each of the two stimulus types that subtended this level of attentional control), 57 LOW, and 66 simple bold arrows (sensorimotor control condition). The total duration of the task was 10 min and 8 s. A fixation cross-hair was presented during the inter-stimulus interval, which ranged from 2,000 to 6000 ms. Stimuli were presented using a back-projection system, and responses were recorded through a fiber-optic response box that allowed the accuracy and reaction time to be measured for each trial. In order to maximize the detection of the effects of interest, focus was centered on the analysis of the high level of attentional control elicited by the VAC task.

#### fMRI data acquisition

Blood oxygen level-dependent (BOLD) fMRI was performed on a GE Signa 3T scanner (General Electric, Milwaukee, WI) equipped with a standard quadrature head coil. A gradient-echo planar imaging sequence (repetition time: 2000 ms; echo time: 28 ms; 26 interleaved axial slices; thickness: 4 mm; gap: 1 mm; voxel size: 3.75 isotropic; scan repetition: 300; flip angle: 90°; field of view: 24 cm; matrix: 64 × 64) was used to acquire images while subjects performed the VAC task. The first four scans were discarded to allow for signal saturation. Images for each subject were realigned, spatially normalized into the MNI template (12-parameter affine model), and spatially smoothed (10-mm Gaussian filter). After realignment, datasets were also checked for small-motion correction (<2 mm in translation, <1.5° in rotation).

#### fMRI data processing

fMRI responses were modeled using a canonical hemodynamic response function and temporally filtered using a 128-Hz high-pass filter and an hrf-shape low-pass filter. Vectors were created for each condition using the timing of the correct responses. To account for differences in head movement between groups, residual movement was also modeled as a regressor of no interest. A *t*-statistic was then used to produce a statistical image for BOLD responses relative to the brain processing of stimuli for each level of attentional control (HIGH, INT, and LOW). Group analysis was performed using a general linear model with BOLD response at the high level of attentional control (HIGH) as the dependent variable in order to maximize the effects of interest. The *DRD2* rs1076560 genotype was used as the categorical predictor and SPQ scores as the continuous predictor. Separate contrasts were thus performed to detect the directionality of the correlations (i.e., positive or negative) associated with SPQ scores for each *DRD2* genotype. We used a statistical threshold of *p* < 0.05 which was family-wise error small-volume corrected using regions of interest and created using WFU PickAtlas software version 1.04.^1^ The dorso-lateral pre-frontal cortex (DLPFC; BA9/46) and the anterior cingulate cortex (BA24/32), which are crucially associated with attentional control and are modulated by dopamine signaling (Blasi et al., 2007, 2010, 2011), were considered as regions of interest. Brodmann’s areas were assigned to activated clusters using the Talairach Daemon.^2^ All coordinates are reported in the MNI system.

### Analysis of behavioral data

Multiple regression was performed to investigate the behavioral performance on the VAC. In particular, accuracy or reaction time at the high level of attentional control (HIGH) was used as the dependent variables, *DRD2* rs1076560 was used as the categorical predictor, and SPQ scores were the continuous predictor.

## Results

### SPQ and *DRD2* rs1076560

Genotype groups were matched for age (*F* = 1.4, *p* = 0.3), IQ (*F* = 0.37, *p* = 0.5), handedness (*F* = 1.82, *p* = 0.2), and gender (χ^2^ = 3.6, *p* = 0.1). The one-way ANOVA with the SPQ total score as the dependent variable and the *DRD2* rs1076560 genotype as the predictor showed a main effect of the genotype on SPQ scores (*F*_1,65_ = 7.5, *p* = 0.008), with GT individuals having greater scores than GG subjects.

### SPQ, *DRD2* rs1076560 and IBZM binding

**Figure 1:**
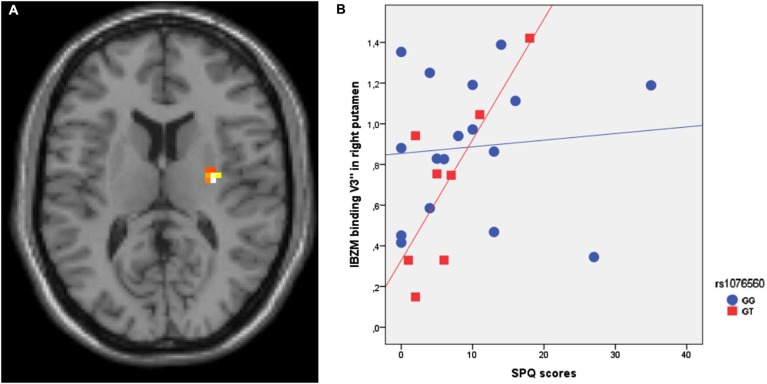
**(A)** Axial section of the brain showing the interaction between rs1076560 and SPQ scores on IBZM binding V3″ in the right putamen. **(B)** IBZM binding V3″ extracted from the right putamen cluster illustrated in **(A)**.

SPM multiple regression analysis was performed to investigate the association of IBZM binding in healthy individuals with SPQ scores *per se* and as a function of rs1076560 genotype. Genotype groups were matched for age (*F* = 0.2, *p* = 0.7), IQ (*F* = 0.75, *p* = 0.4), handedness (*F* = 1.3, *p* = 0.3), and gender (χ^2^ = 0.17, *p* = 0.7). SPM multiple regression analysis between IBZM binding as the dependent variable, the *DRD2* rs1076560 genotype as the categorical predictor, and the SPQ score as the continuous predictor indicated an interaction between SPQ scores and genotype in the right putamen (*x*:30; *y*:−12; *z*:12; *z* = 4.67, *p* = 0.001) (Figure 1A). In particular, a positive regression between IBZM binding and SPQ scores was present in GT individuals (*z* = 4.81, *p* = 0.001), while there was no significant correlation in GG subjects (Figure 1B).

### SPQ, *DRD2* rs1076560 and brain activity during attentional control

**Figure 2:**
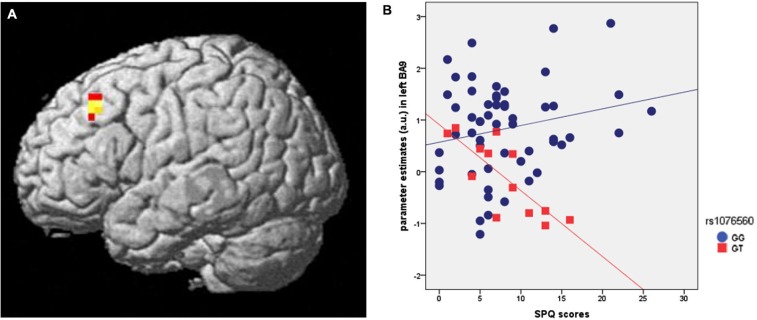
**(A)** Rendered image of the brain showing the interaction between rs1076560 and SPQ scores on fMRI response during attentional control in left BA9 (*x*:−44, *y*:27, *z*:40). **(B)** Parameter estimates extracted from the cluster in BA9 cluster illustrated in **(A)**.

Genotype groups were matched for age (*F* = 3.3, *p* = 0.1), IQ (*F* = 0.5, *p* = 0.5), handedness (*F* = 0.4, *p* = 0.5) and gender (χ^2^ = 0.14, *p* = 0.7). SPM multiple regression analysis was performed to investigate if brain activity during attentional control in healthy individuals was predicted by SPQ scores *per se* and as a function of rs1076560 genotype. This analysis indicated an interaction between SPQ scores and rs1076560 on DLPFC activity (*x*:−44, *y*:27, *z*:40; *z* = 3.4, *p* = 0.028; BA9) (Figure 2A). In particular, a negative regression between dorsolateral prefrontal activity during the high load of attentional control elicited by the VAC task and SPQ scores was present in GT subjects (*z* = 3.80, *p* = 0.009), while there was no significant correlation in GG individuals (Figure 2B). No other significant results were observed in different ROIs such as BA46, BA32 and BA24.

### SPQ, *DRD2* rs1076560 and behavioral performance on the VAC

**Figure 3:**
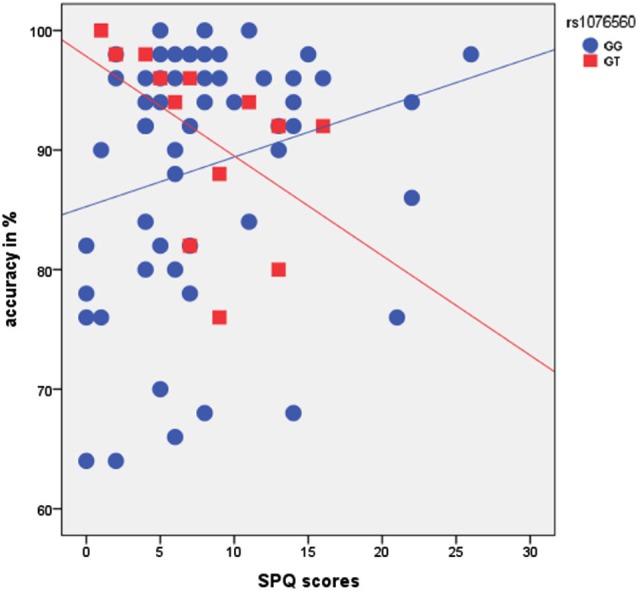
**Scatterplot showing the relationship between rs1076560, SPQ scores, and % accuracy in the VAC task**. See text for statistics.

There was a main effect of rs1076560 (*F* = 4.3, *p* = 0.04), no main effect of SPQ (*F* = 0.4, *p* = 0.5), and a statistical trend for an interaction between rs1076560 and SPQ (*F* = 3.5, *p* = 0.06) on accuracy with a high load of the VAC task. Furthermore, there was a trend toward significance of the entire model (multiple *R* = 0.3, multiple *R*^2^ = 0.08, adjusted *R*^2^ = 0.04, *F* = 1.98, *p* = 0.1). In particular, a negative relationship between behavioral performance in terms of accuracy at high load of the VAC task and SPQ scores was present in GT subjects, while this relationship was not present in GG individuals (Figure 3). We did not find a main effect of rs1076560 and SPQ or their interaction on the VAC task high-load reaction time (All *F* < 0.6, *p* > 0.4). Also, a regression model with rs1076560 and SPQ as regressors and high-load reaction time as the dependent variable did not show any effect as a whole (multiple *R* = 0.12, multiple *R*^2^ = 0.013, adjusted *R*^2^ = −0.03, *F* = 0.29, *p* = 0.8).

## Discussion

We investigated whether schizotypy is related to genetic variation within the *DRD2* gene and whether they interact on a series of phenotypes implicated in schizophrenia. We found that *DRD2* rs1076560 is associated with SPQ scores in healthy subjects. Furthermore, we report an interaction between SPQ scores and the rs1076560 genotype on striatal IBZM binding. This interaction is also present on prefrontal activity during attentional processing. Interaction between SPQ scores and genotype is coherently evident on attentional performance at the behavioral level, but it does not reach full statistical significance.

Previous studies have found higher measures of schizotypy in first-degree relatives of patients with schizophrenia compared with healthy subjects (Kety et al., 1994; Kendler et al., 1995; Tsuang et al., 1999; Vollema et al., 2002), suggesting that this trait is related to risk for this disorder. Our findings indicate that rs1076560 GT subjects have greater SPQ scores than GG individuals and are in line with previous reports suggesting that the T allele increases risk for several biological phenotypes associated with schizophrenia, including inefficient cortical and subcortical responses during cognitive processing (Zhang et al., 2007; Bertolino et al., 2010; Blasi et al., 2011), as well as low “pre-” and “post-” synaptic D2 receptor availability, possibly reflecting greater levels of striatal dopamine signaling (Bertolino et al., 2010).

Previous radiotracer imaging studies indicated that the steady-state and stimulus-induced release of dopamine in the striatum and D2 binding are associated with schizotypy (Abi-Dargham et al., 2004; Soliman et al., 2008; Woodward et al., 2011; Chen et al., 2012). Consistently, we report an interaction between SPQ scores and the rs1076560 genotype on striatal IBZM binding. Our results demonstrate that only GT subjects show a positive correlation between IBZM binding in the right putamen and SPQ scores. Such a correlation was not found in GG individuals. Even though the specificity of IBZM binding for the two D2 isoforms is still uncertain, IBZM is believed to bind post-synaptic D2 receptors (Kung et al., 1990; Nyberg et al., 2009). Therefore, our results suggest that individuals carrying the T allele with greater schizotypy scores have a greater post-synaptic D2 receptor density. Notably, the T allele group is also associated with a lower ratio of expression between D2S and D2L. Together with previous evidence indicating that the T allele of rs1076560 is a risk factor for phenotypes associated with schizophrenia (Zhang et al., 2007; Bertolino et al., 2009a,b, 2010; Blasi et al., 2009, 2011; Fazio et al., 2011), this knowledge suggests that schizotypy in healthy subjects may be mapped onto the Gaussian distribution of schizophrenia risk.

Previous studies have demonstrated that higher-order cognition and related prefrontal activity are strongly modulated by cortical and subcortical dopamine D2 signaling and related genetic variation (Zhang et al., 2007; Tan et al., 2008; Bertolino et al., 2009a). Our fMRI results are consistent with these findings and provide further evidence that *DRD2* functional variation interacts with schizotypy in modulating the attention-related prefrontal response. In particular, we found a negative correlation between behavioral performance, prefrontal activity during attentional processing, and SPQ scores only in GT individuals. In other words, higher schizotypy scores in this subgroup of individuals are related to lower prefrontal activity during attentional control. Similar results were found when looking at the interaction between SPQ scores and the rs1076560 genotype on behavioral accuracy in the VAC task. There was a statistical trend suggesting that higher SPQ scores predict lower accuracy in the task in GT individuals only. The fact that this finding does not reach statistical significance is consistent with the greater biological gap between gene effects and behavior compared with physiological responses, thus requiring greater sample sizes to detect significant effects.

Altogether, these fMRI and behavioral results add evidence to the relevance of the interaction between this construct of personality and genetic variation in *DRD2* in modulating brain physiology during attentional processes. Additionally, they are again consistent with the relationship between schizotypy in healthy subjects and the schizophrenia continuum. Consistently, previous studies have indicated that patients with schizophrenia have abnormal prefrontal activity and worse behavioral performance during attentional processing (Weiss et al., 2003; Honey et al., 2005; Kerns et al., 2005; Laurens et al., 2005; MacDonald et al., 2005; Gur et al., 2007). Furthermore, evidence of abnormal prefrontal activity has been reported during cognitive processing in patients with schizophrenia carrying the rs1076560 T allele (Bertolino et al., 2009a,b, 2010; Blasi et al., 2010).

A limitation of this study is that the sample used for fMRI, SPECT, and SPQ investigations do not overlap. Another limitation is that we investigated the scores of schizotypy in healthy subjects, thus leaving the relationship between schizophrenia as a diagnostic category and the present findings an open question. However, our results are consistent overall in indicating the relevance of the interaction between schizotypy and rs1076560 for the phenotypes measured in the present study.

In conclusion, we have provided evidence for a modulation of imaging phenotypes of relevance to schizophrenia by the interaction between complex personality traits and simple genetic variations. These findings open new scenarios for the study of sub-threshold correlates adding risk for this brain disorder.

## Conflict of interest statement

Prof. Alessandro Bertolino is a full time employee of Hoffman-La Roche Ltd.

## Footnotes

^1^http://fmri.wfubmc.edu/cms/software#PickAtlas

^2^http://www.talairach.org/daemon.html
